# Racial and Ethnic Disparities and the National Burden of COVID-19 on Inpatient Hospitalizations: A Retrospective Study in the United States in the Year 2020

**DOI:** 10.1007/s40615-024-02069-y

**Published:** 2024-09-24

**Authors:** Amanda Nguyen, Russell G. Buhr, Gregg C. Fonarow, Jeffrey J. Hsu, Arleen F. Brown, Boback Ziaeian

**Affiliations:** 1 Department of Medicine, UC Davis Medical Center, Sacramento, CA USA; 2 Division of Pulmonary, Critical Care and Sleep Medicine, David Geffen School of Medicine at UCLA, Los Angeles, CA USA; 3 Greater Los Angeles Veterans Affairs Health Care System, Department of Medicine, Los Angeles, CA USA; 4 Division of Cardiology, David Geffen School of Medicine at UCLA, Los Angeles, CA USA; 5 Division of Cardiology, VA Greater Los Angeles Healthcare System, 1301 Wilshire Blvd, 111E, Los Angeles, CA 90073 USA; 6 Division of General Internal Medicine and Health Services Research, Department of Medicine, David Geffen School of Medicine at UCLA, Los Angeles, CA USA

**Keywords:** Disparities, Race, Ethnicity, COVID-19, Disease burden

## Abstract

**Background:**

Since January 2020, COVID-19 has affected more than 100 million people in the U.S. Previous studies on racial and ethnic disparities related to characteristics and outcomes of COVID-19 patients have been insightful. However, appropriate epidemiologic age-standardization of the disease burden and disparities for hospitalization data are lacking.

**Objective:**

To identify and describe racial and ethnic disparities for primary COVID-19 hospitalizations in the U.S. in 2020.

**Methods:**

In this nationally representative observational study, we use the National Inpatient Sample to quantify racial and ethnic disparities in COVID-19 hospitalizations. Descriptive statistics for patient characteristics, common comorbidities, age-standardized hospitalization rates, inpatient complications, and mortality among COVID-19 hospitalizations were contrasted by race and ethnicity.

**Results:**

There were 1,058,815 primary COVID-19 hospitalizations in 2020. Of those, 47.2% were female, with median age of 66 (IQR, 54, 77). Overall inpatient mortality rate was 11.1%. When compared to White patients, Black, Hispanic, and Native American patients had higher age-standardized hospitalization rate ratios of 2.42 (95% CI 2.40–2.43), 2.26 (2.25–2.28), and 2.51 (2.46–2.56), respectively. Non-White patients had increased age-adjusted rates for procedures and complications. Factors associated with inpatient mortality include age, male sex, Hispanic or Native American race or ethnicity, lower income, Medicaid, heart failure, arrhythmias, coagulopathy, and chronic liver disease.

**Conclusions:**

Marginalized populations in the U.S. had over twice the COVID-19 hospitalization rate relative to White patients. Age-adjusted mortality rates were highest for Black, Hispanic, and Native American patients. Careful consideration for vulnerable populations is encouraged during highly communicable respiratory pandemics.

**Supplementary Information:**

The online version contains supplementary material available at 10.1007/s40615-024-02069-y.

## Introduction

The first reported case of a pneumonia-like illness later identified as COVID-19 occurred in December 2019 in Wuhan, China [1]. Since January 2020, there have been over 770 million cases of COVID-19 across the world, with more than 100 million confirmed cases in the U.S. [2]. These cases have resulted in over 6 million COVID-19 hospitalizations, and over 1 million COVID-19 deaths in the U.S. to date [3]. Recent studies on patient care and outcomes of COVID-19 patients are providing insight into the national burden that COVID-19 had in the U.S. [4–9]. As with many medical conditions, healthcare and socioeconomic disparities disproportionately affect marginalized populations and put them at higher risk for morbidity and mortality [10–13]. As highlighted by the CDC, interpersonal and structural racism negatively affects marginalized groups and is pervasive among all aspects of their health and potential [14]. Published research finds Black, Native American, and Hispanic patients were at higher risk for adverse outcomes and mortality related to COVID-19 [4–9]. However, as populations in different race and ethnic groups may have varying age distributions, the risk of disease and associated outcomes can differ by age [15]. In particular, marginalized race and ethnic groups in the U.S. tend to have younger age distributions than their White counterparts [16, 17]. Thus, appropriately age-standardized national estimates are still needed in order to remove confounding related to age. This allows for more accurate comparisons of COVID-19 hospitalization and mortality rates, as well as other outcomes and complications, between different race and ethnic groups.

To better understand the national health impact of the first year of the COVID-19 pandemic and health equity in treatment access and outcomes, we use nationally representative data standardized to the 2000 U.S. standard population to evaluate age-adjusted racial and ethnic disparities in COVID-19 hospitalizations, comorbid risk factors, hospital procedures performed, and inpatient outcomes. Our analysis adds to the current literature by providing in-depth detail regarding appropriately age-standardized outcomes, procedures, and complications of COVID-19 hospitalizations among distinct race and ethnic groups.

## Methods

### Study Design and Database

This study is a cross-sectional observational study using nationally representative data from the 2020 National Inpatient Sample (NIS). As part of the Healthcare Research and Quality Healthcare Cost and Utilization Project (HCUP), the NIS is a publicly available de-identified database containing information on healthcare utilization, cost, and outcomes for inpatient hospitalization. The NIS database samples inpatient data from 20% of participating hospitals across the nation, and is able to estimate > 97% of all U.S. hospitalizations [18]. All estimations and analyses were performed using the recommended NIS trend weights [19]. NIS data from the year 2020 is the first nationally representative all-payer database to provide a perspective on events and outcomes related to the COVID-19 pandemic in U.S. hospitals. The NIS does not account for readmissions and each observation is coded as a unique hospitalization. HCUP databases, including the Clinical Classifications Software Refined and Elixhauser Comorbidity Software Refined databases, were used to group ICD-10 diagnostic and procedure codes into meaningful categories for analysis (Supplementary Table 1) [20, 21].

### Study Population

All COVID-19 hospitalizations for children and adults ages 0 to 124 from the NIS database from the year 2020 were included. COVID-19 hospitalizations were further characterized as primary COVID-19 hospitalizations if the first listed discharge code (ICD-10 Code U07.1) was for COVID-19, and secondary COVID-19 hospitalizations if COVID-19 was included in any hospitalization code excluding the primary position. COVID-19 hospitalizations were categorized as primary versus secondary hospitalizations in an attempt to distinguish hospitalizations that were primarily affected by COVID-19 as opposed to another primary diagnosis in which the patient was concurrently diagnosed with COVID-19. NIS race and ethnicity categories used include non-Hispanic White, non-Hispanic Black, Hispanic, non-Hispanic Asian-American and Pacific Islander (AAPI), non-Hispanic Native American, and non-Hispanic Other. As defined by HCUP, other race and ethnicity include patients who identify with multiple races or race and ethnicity other than White, Black, Hispanic, AAPI, and Native American. If data on race and ethnicity were available for each observation in the NIS database, ethnicity took precedence over race. Data from missing race and ethnicity was excluded from the analysis [22].

The CDC created a COVID-19 hospitalization surveillance network (COVID-NET) to track numbers and rates of COVID-19 hospitalizations on March 1, 2020 [1, 23]. We excluded COVID-19 codes prior to March 1, 2020, as they were likely coding errors.

### Outcomes

The primary outcome of this study was to estimate disparities in the national age-standardized COVID-19 hospitalization rates, complications, medical procedures, and mortality rates by race and ethnicity.

Secondary outcomes included investigating disparities in baseline patient and hospital characteristics, comorbid conditions, and disposition among COVID-19 hospitalizations. Data on median income was estimated by neighborhood zip code provided by the NIS database. To estimate hospitalization costs, we utilized the total charges and adjusted by cost-to-charge ratio, as supplied by HCUP. All endpoints assessed in this study were analyzed for COVID-19 hospitalizations utilizing ICD-10 diagnostic and procedure codes for outcomes of interest. All ICD-10 codes utilized in this study are listed in Supplementary Table 1.

### Statistical Analysis

Descriptive statistics are reported for patient and hospital characteristics, common comorbidities, and inpatient mortality among COVID-19 hospitalizations. Direct standardization with single-year of life age adjustments was used to age-adjust rates relative to the 2000 U.S. standard population, as recommended by the National Center for Health Statistics. Race and ethnicity data from the 2020 United States Census Bureau was further used to calculate crude and age-standardized hospitalization rates per 100,000 people. The race and ethnicity categories extracted from the Census Bureau correlated to those defined by HCUP, and included Hispanic, non-Hispanic White, non-Hispanic Black, non-Hispanic AAPI, non-Hispanic Native American, and non-Hispanic Other, which consists of patients of multiple races or race/ethnicity other than White, Black, Hispanic, AAPI, and Native American [24]. Standardized hospitalization rate ratios and excess rates for the race and ethnic subgroups were estimated, utilizing White patients as the reference group. In particular, rate ratio was defined as the ratio of age-standardized hospitalization rate of the race/ethnic subgroup, compared to White patients. Excess hospitalization rate was defined as the absolute difference of age-standardized hospitalization rate between the race/ethnic subgroup, compared to White patients. Direct age-standardization was calculated to remove confounding related to age, allowing for more accurate comparisons between different race and ethnic groups. We used multivariable logistic regression using a restricted cubic spline model to adjust for age to evaluate the independent association of prespecified risk factors, such as insurance status, median income level, race and ethnicity, hospital location/division, and comorbid conditions, on inpatient mortality among COVID-19 hospitalizations [25]. All estimations were performed with appropriate NIS survey weights and cluster adjustment to account for the sampling strategy. All statistical analyses were done in Stata 18.0 (StataCorp, College Station, TX).

The UCLA institutional review board granted an oversight exemption given the use of de-identified, public data.

## Results

### Demographic and Clinical Characteristics Associated with COVID-19 Hospitalizations

In the 2020 NIS, there were 1,058,815 primary COVID-19 hospitalizations in children and adults ages 0 to 124, of which 8775 were pediatric hospitalizations, and 1,050,040 were adult hospitalizations. There were 1030 primary COVID hospitalizations that were excluded due to missing demographic data. Of those hospitalized with primary COVID-19, 47.2% were female. With respect to race and ethnicity, 52.5% of patients were White, 20.7% Hispanic, 18.5% Black, 4.1% other race, 3.2% AAPI, and 1.0% Native American. The median age was 66 (IQR: 54, 77). White patients admitted for primary COVID-19 tended to be older than those from other race and ethnic groups. Overall, most primary COVID-19 hospitalizations occurred in the south (Table 1). Native American and Black patients had lower median income compared to other race and ethnic groups. Among the race and ethnic groups, AAPI patients with primary COVID-19 had the highest proportion of patients with private insurance, whereas White and Native American patients with primary COVID-19 had the highest proportion of patients with public insurance (Table 1). The most common comorbidities included hypertension, diabetes, and dyslipidemia. Heart failure, atrial fibrillation/flutter, coronary artery disease, chronic kidney disease, and chronic obstructive pulmonary disease (COPD) were also prevalent. Among the different race and ethnic groups, prevalence of these comorbidities varied (Table 1).

**Table 1:** Baseline patient characteristics/demographics and hospital characteristics for primary COVID-19, by race and ethnicity

Characteristic	All patients	White	Black	Hispanic	AAPI	Native American	Other
	*n* = 1,058,815^a^	*n* = 538,490	*n* = 189,865	*n* = 212,235	*n* = 33,080	*n* = 10,695	*n* = 42,175
Median age, years (IQR 25%, 75%)	66 (54, 77)	71 (60, 80)	62 (51, 72)	58 (46, 70)	64 (52, 75)	59 (46, 70)	62 (49, 73)
**Sex**							
Female	47.2%	47.0%	53.3%	43.7%	46.1%	51.7%	41.9%
**Median income quartile**							
Quartile 1 ($1–49,999)	34.3%	27.6%	52.3%	37.8%	15.4%	58.4%	27.5%
Quartile 2 ($50,000–64,999)	27.7%	30.0%	22.7%	27.2%	23.4%	22.3%	25.1%
Quartile 3 ($65,000–85,999)	21.9%	23.8%	15.2%	22.7%	28.3%	12.8%	23.5%
Quartile 4 ($86,000 +)	16.2%	18.7%	9.8%	12.2%	32.9%	6.5%	23.8%
**Insurance status**							
Medicare	51.9%	63.2%	47.1%	33.4%	38.9%	41.1%	37.8%
Medicaid	12.1%	5.4%	15.3%	23.5%	16.8%	23.7%	19.0%
Private insurance	27.7%	26.2%	29.1%	28.5%	36.7%	22.3%	30.1%
Self-pay or no charge	3.6%	1.7%	3.7%	7.8%	3.6%	2.5%	5.9%
Other	4.7%	3.5%	4.8%	6.7%	4.0%	10.4%	7.1%
**Hospital location/teaching status**							
Rural	11.7%	16.3%	8.5%	3.9%	2.2%	21.9%	4.1%
Urban, nonteaching	19.2%	20.9%	14.4%	20.7%	18.2%	15.3%	16.6%
Urban, teaching	69.2%	62.8%	77.1%	75.4%	79.6%	62.8%	79.2%
**Hospital division**							
New England	3.7%	4.2%	2.6%	3.6%	4.1%	0.65%	2.7%
Middle Atlantic	14.0%	12.6%	15.3%	13.9%	19.5%	2.5%	31.5%
East North Central	16.2%	20.4%	16.5%	7.9%	11.4%	5.8%	9.1%
West North Central	7.1%	9.6%	3.4%	1.8%	4.9%	12.9%	2.3%
South Atlantic	20.4%	18.3%	33.5%	17.8%	9.7%	8.0%	17.7%
East South Central	7.1%	9.2%	10.8%	1.1%	1.2%	2.7%	1.4%
West South Central	14.4%	13.1%	13.1%	19.3%	7.3%	10.9%	16.9%
Mountain	6.8%	6.7%	1.6%	9.1%	8.5%	50.3%	6.6%
Pacific	10.4%	5.8%	3.2%	25.5%	33.3%	6.3%	11.8%
**Hospital size**							
Small	25.6%	26.9%	24.7%	23.2%	22.8%	24.2%	21.8%
Medium	28.9%	28.6%	27.8%	30.3%	28.7%	26.8%	33.2%
Large	45.5%	44.4%	47.5%	46.5%	48.5%	49.0%	45.0%
**Comorbidities**							
Any heart failure	16.1%	18.8%	18.7%	9.3%	10.3%	13.7%	10.6%
Arrhythmia							
Atrial fibrillation/flutter	15.1%	20.7%	10.3%	7.3%	10.4%	8.0%	9.7%
Ventricular tachycardia	1.6%	1.7%	2.0%	1.1%	1.4%	1.6%	0.9%
Coronary artery disease	19.8%	24.9%	16.9%	11.8%	15.4%	13.9%	14.4%
Any cerebrovascular disease	3.7%	3.7%	5.2%	2.6%	4.2%	2.5%	3.4%
Peripheral vascular disease	3.9%	4.9%	3.1%	2.4%	3.9%	2.2%	2.4%
Obesity	27.8%	26.2%	34.1%	29.1%	14.9%	32.5%	22.9%
Dyslipidemia	42.0%	47.0%	39.5%	33.6%	44.5%	30.9%	35.7%
Diabetes	40.6%	36.0%	47.7%	44.6%	45.5%	53.7%	41.4%
Hypertension	43.2%	43.8%	45.5%	39.8%	44.6%	38.0%	41.6%
Asthma	8.6%	7.8%	11.2%	8.6%	9.7%	11.9%	7.9%
COPD	15.0%	20.4%	13.1%	5.9%	7.3%	9.5%	8.3%
Sleep apnea	10.3%	12.8%	10.5%	5.7%	4.9%	8.2%	5.0%
Chronic kidney disease	20.1%	20.6%	27.0%	14.3%	18.1%	18.7%	15.1%
Chronic liver failure	0.27%	0.24%	0.17%	0.41%	0.18%	0.65%	0.24%
Thyroid disorders	14.3%	18.7%	8.3%	10.0%	9.8%	11.2%	10.8%
Rheumatoid arthritis	2.0%	2.5%	1.9%	1.3%	0.92%	2.6%	1.4%
Autoimmune disorders	3.4%	4.0%	3.3%	2.3%	1.9%	3.7%	2.3%
HIV/AIDS	0.56%	0.18%	1.7%	0.57%	0.15%	0.19%	0.76%
Immunity disorder/long-term immunosuppression	3.0%	3.5%	3.2%	2.0%	1.9%	1.9%	1.8%
Coagulopathy	12.3%	12.6%	12.0%	11.9%	12.9%	13.5%	12.2%
Hematologic cancer	1.5%	1.9%	1.2%	0.88%	0.79%	0.65%	1.2%
Solid cancer	3.2%	3.4%	3.2%	2.6%	2.9%	2.5%	2.9%
Tobacco use disorder	21.3%	26.7%	18.0%	13.5%	14.0%	17.9%	14.0%
Alcohol use disorder	1.4%	1.4%	1.5%	1.5%	0.67%	5.6%	1.4%
Substance use disorder	1.7%	1.5%	2.6%	1.5%	1.0%	3.7%	1.4%

^a^*N* reported with appropriate NIS trend weights

### Characteristics of Hospital Stays for COVID-19

The mean length of stay was 7.5 days (95% CI 7.4–7.5). Hispanic, Native American, and Other patients had longer mean lengths of stay (Table 2). Hispanic and Other patients admitted for primary COVID-19 had the highest mean cost of stay (Table 2). Total inpatient cost for primary COVID-19 hospitalizations from March 2020 through December 2020 was $20,033,334,264. Only 54.9% of all hospitalized patients were discharged home, with 16.6% being transferred to another care facility such as a skilled nursing facility, and 13.3% requiring home healthcare. Age-adjusted rates used for comparison between race and ethnic groups showed that Native American patients had the least age-adjusted proportion of discharges home, the greatest age-adjusted proportion of discharges to a short-term hospital, and the greatest age-adjusted proportion of discharges against medical advice. AAPI patients had the greatest age-adjusted proportion of discharges home and the lowest age-adjusted proportion of discharges against medical advice. White patients had the greatest age-adjusted proportion of discharges to a skilled nursing facility. Black patients had the greatest age-adjusted rates of patients requiring home healthcare (Table 2).

**Table 2:** Hospital procedures, complications, and disposition for primary COVID-19 by race and ethnicity, unadjusted (age-adjusted)^a^

Characteristic	Overall population	White	Black	Hispanic	AAPI	Native American	Other
	*n* = 1,058,815	*n* = 538,490	*n* = 189,865	*n* = 212,235	*n* = 33,080	*n* = 10,695	*n* = 42,175
**Complications**							
Acute MI	3.4% (1.3%)	3.6% (1.2%)	3.4% (1.6%)	3.0% (1.3%)	3.7% (1.8%)	3.5% (2.0%)	3.4% (1.4%)
Cardiac arrest	1.8% (1.1%)	1.5% (0.86%)	2.4% (1.8%)	2.3% (1.1%)	1.8% (0.86%)	2.1% (2.0%)	2.2% (1.1%)
Acute renal failure	25.2% (14.0%)	24.4% (12.3%)	35.0% (21.7%)	19.4% (11.1%)	23.3% (12.3%)	19.8% (14.3%)	22.7% (12.2%)
Acute liver failure	0.63% (0.49%)	0.50% (0.73%)	0.72% (0.40%)	0.80% (0.55%)	0.82% (0.32%)	0.65% (0.33%)	0.85% (0.66%)
Acute cerebrovascular event	1.7% (0.85%)	1.8% (0.73%)	1.7% (1.3%)	1.3% (0.73%)	1.9% (0.89%)	1.2% (0.83%)	1.8% (0.80%)
Acute thromboembolic events	4.4% (3.2%)	4.3% (3.4%)	5.3% (4.4%)	3.8% (2.6%)	3.2% (2.0%)	4.3% (2.7%)	4.2% (3.1%)
Acute respiratory failure	58.0% (45.7%)	57.1% (45.2%)	52.0% (40.9%)	63.3% (48.6%)	63.5% (49.6%)	67.9% (64.5%)	62.1% (47.2%)
Shock	2.0% (3.1%)	1.7% (4.0%)	2.1% (3.3%)	2.4% (2.7%)	2.3% (1.6%)	2.9% (3.0%)	2.4% (3.7%)
Critical illness myopathy/neuropathy	0.57% (0.41%)	0.52% (0.34%)	0.57% (0.38%)	0.62% (0.44%)	0.56% (0.33%)	1.1% (0.94%)	0.78% (0.42%)
Delirium/disorientation	2.3% (1.0%)	2.7% (1.0%)	1.8% (1.0%)	1.6% (1.0%)	2.2% (0.91%)	2.2% (1.3%)	2.0% (0.79%)
**Hospital procedures**							
Cardiac catheterization	0.16% (0.11%)	0.17% (0.13%)	0.15% (0.10%)	0.12% (0.07%)	0.18% (0.09%)	0.28% (0.17%)	0.17% (0.33%)
CPR	1.4% (0.91%)	1.0% (0.69%)	2.0% (1.6%)	1.8% (0.96%)	1.6% (0.76%)	0.94% (0.74%)	1.6% 0.84%)
Cardioversion	0.34% (0.19%)	0.31% (0.16%)	0.42% (0.22%)	0.31% (0.15%)	0.35% (0.28%)	0.47% (0.45%)	0.30% (0.16%)
Vasopressor infusion	1.8% (1.7%)	1.4% (1.3%)	2.0% (2.0%)	2.4% (1.9%)	2.9% (2.7%)	1.6% (0.84%)	2.1% (1.3%)
MCS/ECMO	0.22% (0.44%)	0.15% (0.36%)	0.24% (0.45%)	0.35% (0.35%)	0.15% (0.12%)	0.37% (0.56%)	0.27% (0.28%)
Noninvasive ventilation	6.4% (5.1%)	6.6% (4.7%)	5.7% (5.2%)	6.2% (5.3%)	6.5% (4.9%)	7.8% (7.2%)	6.1% (4.6%)
Any mechanical ventilation^b^	9.9% (8.0%)	8.6% (8.1%)	10.6% (9.0%)	11.6% (8.3%)	10.7% (6.8%)	15.6% (12.6%)	11.6% (8.1%)
Thoracentesis/thoracoscopic pleural drainage	1.2% (0.79%)	1.1% (0.85%)	0.80% (0.72%)	1.5% (1.0%)	1.2% (0.56%)	2.0% (1.7%)	1.3% (0.86%)
Bronchoscopy	0.82% (0.67%)	0.71% (0.68%)	0.81% (0.56%)	1.0% (0.71%)	0.67% (1.0%)	2.0% (1.7%)	1.1% (0.63%)
Any hemodialysis	4.1% (2.7%)	2.5% (1.5%)	7.4% (4.4%)	5.0% (2.9%)	4.8% (2.8%)	7.9% (6.2%)	4.0% (2.3%)
CRRT	0.61% (0.49%)	0.43% (0.27%)	1.0% (0.72%)	0.70% (0.44%)	0.60% (0.36%)	1.3% (1.8%)	0.50% (0.31%)
Blood transfusion	2.5% (2.6%)	1.9% (1.7%)	3.4% (4.1%)	2.8% (2.5%)	2.8% (1.4%)	2.7% (1.7%)	2.9% (2.0%)
**Hospital stay characteristics**							
Mean length of stay (95% CI)	7.5 (7.4–7.5)	7.1 (7.0–7.2)	7.7 (7.6–7.8)	8.0 (7.8–8.1)	7.6 (7.4–7.9)	8.0 (7.6–8.5)	7.9 (7.7–8.2)
Mean costs (95% CI)	$78,513 ($76,309–$80,716)	$66,920 ($65,224–$68,617)	$74,800 ($71,524–$78,075)	$104,402 ($100,130–$108,674)	$95,042 ($89,168–$100,917)	$83,662 ($73,671–$93,652)	$101,710 ($90,774–$112,646)
**Disposition**							
Home/routine	54.9% (78.8%)	49.0% (78.4%)	56.6% (76.5%)	66.0% (80.4%)	61.1% (80.5%)	60.9% (73.0%)	60.0% (78.9%)
Transfer to short-term hospital	2.9% (2.7%)	3.0% (2.7%)	2.5% (2.4%)	2.8% (2.4%)	3.5% (3.8%)	5.6% (4.7%)	3.1% (2.4%)
Transfer to skilled nursing facility	16.6% (6.3%)	20.7% (7.3%)	15.8% (6.8%)	9.1% (4.7%)	11.5% (4.1%)	10.2% (6.6%)	12.6% (6.2%)
Home healthcare	13.3% (6.8%)	14.8% (6.5%)	13.8% (7.9%)	10.5% (6.8%)	12.5% (6.9%)	7.3% (4.7%)	11.5% (6.6%)
Against medical advice	1.0% (1.3%)	0.81% (1.2%)	1.4% (1.8%)	1.2% (1.1%)	0.65% (0.57%)	1.3% (2.2%)	1.3% (0.92%)
Died in hospital	11.1% (4.2%)	11.7% (3.8%)	9.8% (4.6%)	10.4% (4.7%)	10.6% (4.2%)	14.7% (8.7%)	11.4% (4.9%)

^a^Age-adjusted rates reported in parentheses

^b^Any mechanical ventilation defined as combined mechanical ventilation, intubation, and tracheostomy

### Hospital Complications and Procedures in COVID-19 Hospitalizations

Acute renal failure, acute thromboembolic events, and acute respiratory failure, requiring noninvasive and/or mechanical ventilation, were the most common inpatient complications. Black patients with primary COVID-19 had the highest age-adjusted rates for acute renal failure, acute cerebrovascular event, and acute thromboembolic event. Native American patients had the highest age-adjusted rates of acute myocardial infarction (MI), cardiac arrest, and acute respiratory failure. AAPI patients had the lowest age-adjusted rates of acute liver failure, acute thromboembolic event, and shock (Table 2). Native American patients with primary COVID-19 also had higher age-adjusted procedure rates, including mechanical ventilation, mechanical circulatory support/extracorporeal membrane oxygenation (MCS/ECMO), cardioversion, hemodialysis, and continuous renal replacement therapy (CRRT) compared to other race and ethnic groups. Black patients had the highest age-adjusted rates of cardiopulmonary resuscitation (CPR) and blood transfusions. AAPI patients had the highest age-adjusted rates of vasopressor infusion (Table 2).

### Age-Adjusted Incidence of Hospitalization and In-Hospital Mortality

Throughout 2020, there was a general uptrend in the number of COVID-19 admissions and downtrend in inpatient deaths with hospitalizations occurring in early 2020 exhibiting higher rates of inpatient mortality (Supplementary Fig. 1). Males had a higher age-adjusted COVID-19 hospitalization rate, at 313.3 per 100,000 than females with 237.8 per 100,000. Black, Hispanic, and Native American patients had higher crude and age-adjusted primary COVID-19 hospitalization rates compared to White patients (Table 3).

**Table 3:** Disparity measures of the age-standardized hospitalization rate for primary COVID-19 by sex, race, and ethnicity

	**Overall population**	**Male**	**Female**
**Total primary COVID-19 hospitalization rate**^**a**^			
Crude hospitalization rate	319.4	343.7	296.0
Age-standardized hospitalization rate (95% CI)	272.4 (271.8–272.9)	313.3 (312.5–314.2)	237.8 (237.1–238.5)
**White**			
Crude hospitalization rate	280.9	301.8	260.5
Age-standardized hospitalization rate (95% CI)	194.1 (193.5–194.6)	224.0 (223.2–224.9)	169.2 (168.5–169.9)
**Black**			
Crude hospitalization rate	475.4	466.3	483.6
Age-standardized hospitalization rate (95% CI)	469.0 (466.9–471.2)	502.2 (498.6–505.7)	445.5 (442.7–448.4)
Standardized rate ratio (95% CI)^b^	2.42 (2.40–2.43)	2.24 (2.22–2.26)	2.63 (2.61–2.65)
Excess (95% CI)^b^	274.95 (272.70–277.21)	278.14 (274.53–281.75)	276.34 (273.42–279.25)
**Hispanic**			
Crude hospitalization rate	341.9	386.4	297.7
Age-standardized hospitalization rate (95% CI)	439.5 (437.6–441.5)	530.3 (527.1–533.5)	362.4 (360.0–364.8)
Standardized rate ratio (95% CI)	2.26 (2.25–2.28)	2.37 (2.35–2.38)	2.14 (2.13–2.16)
Excess (95% CI)	245.44 (243.42–247.46)	306.27 (302.94–309.60)	193.17 (190.67–195.66)
**AAPI**			
Crude hospitalization rate	163.4	185.0	143.9
Age-standardized hospitalization rate (95% CI)	158.8 (157.0–160.5)	191.3 (188.5–194.2)	132.4 (130.3–134.6)
Standardized rate ratio (95% CI)	0.82 (0.81–0.83)	0.85 (0.84–0.87)	0.78 (0.77–0.80)
Excess (95% CI)	− 35.33 (− 37.17 to − 33.50)	− 32.68 (− 35.69 to − 29.66)	− 36.77 (− 39.03 to − 34.51)
**Native american**			
Crude hospitalization rate	475.0	465.3	484.3
Age-standardized hospitalization rate (95% CI)	486.4 (476.9–496.1)	506.1 (491.5–520.9)	472.9 (460.1–486.0)
Standardized rate ratio (95% CI)	2.51 (2.46–2.56)	2.26 (2.19–2.33)	2.80 (2.72–2.87)
Excess (95% CI)	292.33 (282.71–301.95)	282.04 (267.38–296.71)	303.74 (290.81–316.68)
**Other**			
Crude hospitalization rate	276.8	330.4	225.9
Age-standardized hospitalization rate (95% CI)	388.0 (384.1–391.9)	497.9 (491.1–504.7)	298.7 (294.1–303.3)
Standardized rate ratio (95% CI)	2.00 (1.98–2.02)	2.22 (2.19–2.25)	1.77 (1.74–1.79)
Excess (95% CI)	193.90 (189.93–197.86)	273.90 (267.06–280.73)	129.51 (124.84–134.18)

^a^Directly standardized rates (per 100,000)

^b^Values are presented as ratios or excess number of admissions per 100,000. Rate is defined as ratio of age-standardized hospitalization rate of subgroup over reference (White). Excess is defined as absolute difference of age-standardized hospitalization rate between subgroup and reference (White)

Overall inpatient mortality rate was 11.1% (Table 2). Patients of Native American, Hispanic, and other race and ethnicity had higher age-adjusted mortality rates compared to their counterparts, at 8.7%, 4.7%, and 4.9%, respectively (Table 2). Black and AAPI primary COVID-19 patients had similar age-adjusted inpatient mortality rates despite the lower age-adjusted primary COVID-19 hospitalization rate among AAPI patients (Tables 2 and 3). A sub-analysis of crude COVID-19 mortality rates was performed, stratified by median income among the different race and ethnic groups (Supplementary Table 2). Among COVID-19 patients in the lowest median income quartile, Black and Native American patients were found to have the highest proportion of inpatient deaths, while AAPI patients had the lowest proportion of deaths (Supplementary Table 2).

Multivariable logistic regression models using a restricted cubic spline model to adjust for age were created to assess the independent association of prespecified risk factors with inpatient mortality (Table 4). Factors associated with inpatient mortality include male sex, heart failure, atrial fibrillation/flutter, ventricular tachycardia, cerebrovascular disease, coagulopathy, obesity, COPD, and chronic liver disease. Patients with lower income and public health insurance were at increased odds of inpatient death (Table 4). Native American patients were at the highest odds of inpatient mortality relative to White patients after adjusting for other factors and comorbidities (Table 4). Although Black patients had higher age-adjusted primary COVID-19 hospitalization rates compared to White patients, they were at decreased odds of inpatient mortality (Tables 3 and 4). There was a non-linear increasing risk for inpatient mortality with age (Supplementary Fig. 2).

**Table 4:** Patient factors associated with inpatient mortality for primary COVID-19 hospitalizations

Characteristic	aOR	CI	*P*-value
Age	See spline graph^a^		
Female sex	0.74	0.72–0.77	< 0.001
**Race (relative to White)**			
Black	0.91	0.87–0.96	0.001
Hispanic	1.26	1.19–1.33	< 0.001
AAPI	1.10	1.00–1.22	0.052
Native American	2.09	1.75–2.49	< 0.001
Other	1.15	1.05–1.26	0.003
**Insurance (relative to Medicare)**			
Medicaid	1.11	1.04–1.19	0.003
Private insurance	0.94	0.89–1.00	0.052
Self-pay or no charge	1.17	1.01–1.34	0.035
Other	1.29	1.17–1.42	< 0.001
**Median income quartile (relative to quartile 1 ($1–49,999)**			
Quartile 2 ($50,000–64,999)	0.90	0.86–0.94	< 0.001
Quartile 3 ($65,000–85,999)	0.81	0.77–0.85	< 0.001
Quartile 4 ($86,000 +)	0.73	0.69–0.78	< 0.001
**Hospital location/teaching status (relative to rural)**			
Urban, nonteaching	1.14	1.04–1.24	0.005
Urban, teaching	1.24	1.14–1.34	< 0.001
**Hospital division (relative to New England)**			
Middle Atlantic	1.49	1.32–1.68	< 0.001
East North Central	0.97	0.87–1.09	0.640
West North Central	1.14	1.00–1.31	0.052
South Atlantic	1.08	0.96–1.21	0.220
East South Central	1.41	1.23–1.62	< 0.001
West South Central	1.27	1.11–1.45	< 0.001
Mountain	1.24	1.08–1.42	0.002
Pacific	1.35	1.20–1.53	< 0.001
**Month of admission (relative to December)**			
November	1.15	1.10–1.21	< 0.001
October	1.10	1.03–1.17	0.005
September	1.16	1.08–1.25	< 0.001
August	1.09	1.01–1.17	0.019
July	1.23	1.16–1.31	< 0.001
June	1.18	1.10–1.28	< 0.001
May	1.31	1.21–1.41	< 0.001
April	2.07	1.94–2.20	< 0.001
March	2.69	2.46–2.95	< 0.001
**Comorbid conditions**			
Heart failure	1.28	1.22–1.33	< 0.001
Atrial fibrillation/flutter	1.54	1.48–1.60	< 0.001
Ventricular tachycardia	3.23	2.94–3.54	< 0.001
Cerebrovascular disease	1.64	1.54–1.74	< 0.001
Coagulopathy	2.13	2.03–2.23	< 0.001
Obesity	1.34	1.29–1.39	< 0.001
Diabetes mellitus	1.17	1.13–1.21	< 0.001
Hypertension	0.86	0.83–0.90	< 0.001
COPD	1.21	1.16–1.26	< 0.001
Asthma	0.83	0.78–0.89	< 0.001
Tobacco use disorder	0.77	0.74–0.80	< 0.001
Alcohol use disorder	1.03	0.91–1.18	0.619
Drug related disorders	0.89	0.77–1.02	0.100
Chronic kidney disease	1.23	1.18–1.29	< 0.001
Chronic liver disease	3.61	2.96–4.40	< 0.001
HIV/AIDS	1.13	0.88–1.44	0.346
Immunity disorder/long-term immunosuppression	1.16	1.06–1.27	0.001
Autoimmune disorders	1.17	1.07–1.27	< 0.001

^a^See Supplementary Fig. 2 for the spline model for age

### Secondary COVID-19 Hospitalizations

There was a total of 620,180 secondary COVID-19 hospitalizations in children and adults ages 0 to 124 in 2020, with increased mortality rates early on in 2020 (Supplementary Fig. 3). Of those 620,180 secondary COVID-19 hospitalizations, 11,185 were pediatric hospitalizations and 608,995 were adult hospitalizations. There were 1565 secondary COVID hospitalizations that were excluded due to missing demographic data. Total inpatient costs for secondary COVID-19 hospitalizations from March 2020 through December 2020 were $15,773,031,618. Similar trends in patient and hospital characteristics, as well as hospital procedures, complications, and outcomes, were observed for secondary COVID-19 (Supplementary Table 3 for baseline patient/hospital characteristics, Supplementary Table 4 for procedures and outcomes, Supplementary Table 5 for age-standardized hospitalization rates and Supplementary Table 6 for risk factors associated with inpatient mortality).

## Discussion

There have been many early studies, including multi-center studies and systemic reviews, that have reported disparities in COVID-19 hospitalizations and death in non-White patients, particularly among Black and Hispanic patients [26, 27]. Recent publications utilizing the 2020 NIS database have been insightful in describing racial and ethnic disparities using nationally representative data. However, these studies focused primarily on adult hospitalizations and did not utilize direct standardization to adjust for age when comparing different race and ethnic groups [4, 6]. Thus, any confounding bias attributed to age may skew the perceived disparities reported by these studies. Using the COVID-NET database, CDC estimates have utilized direct standardization to report greater age-standardized COVID-19 hospitalization rates among Black, Hispanic, and Native American adult patients [28–30]. However, these estimations were standardized to the 2019 U.S. intercensal population, and not the 2000 U.S. standard population, which is the standard population recommended by the National Center for Health Statistics. To help address this gap in the literature, our study implements direct-standardization of rates among all-age pediatric and adult COVID-19 hospitalizations to estimate age-adjusted rates standardized to the 2000 U.S. standard population. When appropriately standardizing COVID-19 hospitalization rates by race and ethnic national populations, we found Black, Hispanic, and Native American patients had over double the hospitalization rate when compared to the national White population. AAPI patients had roughly four-fifths of the primary COVID-19 hospitalization rate compared to the White national population.

Compared to previous studies, our study also allows a more accurate and detailed comparison of inpatient procedures, complications, and outcomes between race and ethnic groups by adjusting for age [5–7, 27–33]. While crude inpatient mortality was highest for White and Native American patients, age-adjusted mortality rates found White patients had the lowest mortality rate and Native American patients had the highest risk. With appropriate age adjustment, we find that Black, Hispanic, and Native American patients not only had higher hospitalization rates, but also worse outcomes. Many prior studies utilized race groups that combined AAPI, Native American, and other races into one joint group, losing the granularity of detail for outcomes in each of these specific race groups [6, 27]. For example, using data from the 2020 NIS database, Vardar et al. report crude rates of 59.1%, 51.5%, 59.8%, and 60.3% for acute respiratory failure in White, Black, Hispanic, and Other adult patients, respectively [6]. Our study reports similar crude rates of acute respiratory failure for White, Black, Hispanic, AAPI, Native American, and Other patients. However, after adjusting for age, we find that Native American patients were at highest risk of acute respiratory failure at 64.5% compared to 45.7%, 45.2%, 40.9%, 48.6%, 49.6%, and 47.2% for White, Black, Hispanic, AAPI, and Other patients, respectively. We report similar disparities for other age-adjusted inpatient complications and procedures among marginalized communities with COVID-19, as described in Table 2.

As emphasized by the CDC, interpersonal, structural, and systemic racism greatly contribute to health inequities that impact non-White persons in the U.S. Marginalized populations are at increased risk for adverse health outcomes [10–14]. They are at increased risk for chronic comorbid conditions, lower access to healthcare, and lower working and neighborhood living conditions relative to White populations in the U.S. [8]. The large difference in COVID-19 hospitalization burden that we noted in our study likely relates to increased transmission related to employment differences and household and neighborhood characteristics. Working adults without flexible sick leave are more likely to work while sick, and spread transmissible illness among their coworkers, clients, and/or customers [34]. Prior research has suggested that essential workers are at increased risk for COVID-19 infection and transmission due to occupational exposures and the nature of their work. Among non-White individuals, Black and Hispanic individuals are more likely to hold essential worker positions and have been disproportionately impacted by COVID-19 [35–37]. In many marginalized communities, multiple generations live in a single household, putting the young and elderly at increased risk of COVID-19 infection [38]. Access to and usage of high-quality personal protective equipment may also contribute to differences in infection rates between national populations.

Moreover, socioeconomic status is a multi-dimensional concept that is strongly influenced by the social construct of race and racism. Differences in socioeconomic status among different race and ethnic groups in turn play a major role in health disparities [39, 40]. Various studies have suggested that the number of deaths due to social determinants of health, including low education, racial segregation, and income equality, is comparable to the total number of deaths from leading medical conditions, such as acute MI, cerebrovascular disease, and lung cancer [41, 42]. In one study, poverty was demonstrated to be the strongest socioeconomic factor for heart failure mortality and was likely mediated by chronic illnesses such as diabetes and obesity [43]. Although a thorough analysis of socioeconomic factors could not be assessed in our study due to the inherent limitations of the NIS database, we did find that differences in neighborhood household income and Medicaid insurance status were associated with higher odds of inpatient mortality. In our study, AAPI patients were noted to live in higher neighborhood income quartiles, had higher rates of private insurance, and had the lowest age-adjusted rates of inpatient mortality. Furthermore, among COVID-19 patients in the lowest median income quartile, Black, Hispanic, and Native American patients had the highest proportion of deaths, while AAPI patients had the lowest proportion of deaths in this income quartile.

Among the COVID-19 healthcare disparities noted in our study, of particular note is the marked discrepancies demonstrated in Native American patients. As highlighted in previous studies, medical mistrust and lower satisfaction are prevalent among Native American patients in the U.S. [44]. This is partly due to the unfortunate history of displacement of indigenous people from their lands, as well as unethical research among members of their community [45, 46]. These factors may very well be contributing to the marked disparities in COVID-19 hospitalizations demonstrated in our study. As such, an understanding of the history and culture of indigenous patients, as well as further research, is encouraged to address these health inequities.

In conclusion, the large disparities in hospitalization and mortality among different race and ethnic groups during the COVID-19 pandemic were likely driven by many of the same factors of systemic and structural racism that have afflicted marginalized communities for centuries [10–13, 47–50]. Developing more direct pandemic response for marginalized populations is encouraged for all pandemic responses and communicable disease response. Programs and policies should aim at investigating and addressing the root causes for health inequities among vulnerable groups. It is imperative to take into account the history of racism and its contributions in creating structural barriers to education, employment, and health and how this impacts the wellness of individuals and communities as a whole. Future research into the status and availability of COVID-19 vaccinations and therapies among marginalized populations may also be insightful.

## Limitations

This study was performed using nationally representative claims data. Inaccurate administrative coding may weaken the estimates we have provided. Administrative codes do not indicate whether a secondary condition was present on admission or developed during hospitalization. This may overestimate the prevalence of certain inpatient complications or baseline comorbid conditions. In addition, data from the NIS does not account for readmissions. Each observation is coded as a unique hospitalization and may affect the outcomes analyzed in this study. Furthermore, the collection and reporting of race and ethnicity data from the NIS can vary greatly by hospital, from self-identification to presumed identity. Data for the AAPI and other race/ethnic categories as defined by NIS and utilized in this study are limited by the problem of data aggregation of very different populations. Thus, reported differences may be difficult to quantify in a meaningful way. Furthermore, as the sample size for each race and ethnic group assessed in this study varied significantly, outcomes analyzed may be at increased risk of false positives. As the NIS database only provides information on inpatient factors and outcomes, residual measured and unmeasured confounding factors may bias estimates and/or limit the conclusions that can be made from this study. As a result, our study may not fully reflect the potential systemic and structural racism factors that affect COVID-19 health outcomes. Further research into detailed social determinant of health factors affecting COVID-19 outcomes would be beneficial. This study was also completed using data early in the COVID-19 pandemic when patterns of care were rapidly changing and there was limited and shifting evidence on what interventions and treatments were successful.

We tried to identify the impact that COVID-19 had on patients with primary or secondary COVID-19. Initially, we had hypothesized that patients with primary COVID-19 diagnoses may have been more significantly affected by the infection, leading to COVID-19 as the primary diagnosis of their hospitalization. On the other hand, patients who were admitted for a different primary diagnosis and had secondary COVID-19 diagnoses may have experienced more complications and mortality due to underlying acute disease. However, given the variability with administrative coding, our reported data on primary and secondary COVID-19 diagnoses may not be truly reflective of differences between these hospitalizations.

## Conclusions

In summary, marginalized populations in the U.S. faced the largest age-adjusted hospitalization and mortality burden related to the COVID-19 pandemic. Social differences, likely driven by intrinsic, systemic, and structural racism, led to large observable differences in disease transmission and hospitalization outcomes during a novel viral pandemic. Future pandemic planning should strive to integrate direct strategies to better protect vulnerable populations to disrupt viral transmission while new treatments and vaccines are being developed.

## Supplementary Information

Supplementary Fig. 1 Figure showing number of primary COVID-19 admissions and mortality rate, by month.

^a^Mortality rate defined as the number of primary COVID-19 deaths/total number of primary COVID-19 admissions.

Supplementary Fig. 2 Age spline model, showing non-linear relationship between age and odds of inpatient mortality.

Supplementary Fig. 3 Figure showing number of secondary COVID-19 admissions and mortality rate, by month.

^a^Mortality rate defined as the number of secondary COVID-19 deaths/total number of secondary COVID-19 admissions.

## Supplementary Information

Below is the link to the electronic supplementary material.

Supplementary file1 (DOCX 232 KB)

## References

[1] *CDC Museum COVID-19 Timeline*. Centers for disease control and prevention; 2023. Accessed July 5, 2023. https://www.cdc.gov/museum/timeline/covid19.html#:~:text=March%2031%2C%202020&text=One%20of%20the%20first%20warnings,age%2034%20from%20COVID%2D19.

[2] WHO* coronavirus (COVID-19) Dashboard*. World Health Organization Accessed July 5, 2023. https://covid19.who.int/

[3] *COVID data tracker*. Centers for Disease Control and Prevention Accessed July 5, 2023. https://covid.cdc.gov/covid-data-tracker/#datatracker-home

[4] Pal S, Gangu K, Garg I, et al. Gender and race-based health disparities in COVID-19 outcomes among hospitalized patients in the United States: a retrospective analysis of a national Sample. Vaccines. 2022;10(12):2036. 10.3390/vaccines10122036. 36560446 10.3390/vaccines10122036 PMC9781042

[5] Aleligne YK, Appiah D, Ebong IA. Racial disparities in coronavirus disease 2019 (COVID-19) outcomes. Curr Opin Cardiol. 2021;36(3):360–6. 10.1097/HCO.0000000000000847. 33657019 10.1097/HCO.0000000000000847

[6] Vardar U, Ilelaboye A, Murthi M, et al. Racial disparities in patients with COVID-19 infection: a national inpatient sample analysis. *Cureus*. Published online February 15, 2023. 10.7759/cureus.35039 10.7759/cureus.35039 PMC10023870 36942174

[7] Magesh S, John D, Li WT, et al. Disparities in COVID-19 Outcomes by race, ethnicity, and socioeconomic status: a systematic-review and meta-analysis. JAMA Netw Open. 2021;4(11):e2134147. 10.1001/jamanetworkopen.2021.34147. 34762110 10.1001/jamanetworkopen.2021.34147 PMC8586903

[8] Tai DBG, Shah A, Doubeni CA, Sia IG, Wieland ML. The disproportionate impact of COVID-19 on racial and ethnic minorities in the United States. Clin Infect Dis Off Publ Infect Dis Soc Am. 2021;72(4):703–6. 10.1093/cid/ciaa815. 10.1093/cid/ciaa815 PMC7337626 32562416

[9] Webb Hooper M, Marshall V, Pérez-Stable EJ. COVID-19 health disparities and adverse social determinants of health. Behav Med. 2022;48(2):133–40. 10.1080/08964289.2021.1990007. 35318895 10.1080/08964289.2021.1990007

[10] Williams DR, Rucker TD. Understanding and addressing racial disparities in health care. Health Care Financ Rev. 2000;21(4):75–90. 11481746 PMC4194634

[11] Kim Y, Vazquez C, Cubbin C. Socioeconomic disparities in health outcomes in the United States in the late 2010s: results from four national population-based studies. Arch Public Health. 2023;81(1):15. 10.1186/s13690-023-01026-1. 36739440 10.1186/s13690-023-01026-1 PMC9899106

[12] Walker RJ, Strom Williams J, Egede LE. Influence of race, ethnicity and social determinants of health on diabetes outcomes. Am J Med Sci. 2016;351(4):366–73. 10.1016/j.amjms.2016.01.008. 27079342 10.1016/j.amjms.2016.01.008 PMC4834895

[13] Ziaeian B, Kominski GF, Ong MK, Mays VM, Brook RH, Fonarow GC. National differences in trends for heart failure hospitalizations by sex and race/ethnicity. Circ Cardiovasc Qual Outcomes. 2017;10(7):e003552. 10.1161/CIRCOUTCOMES.116.003552. 28655709 10.1161/CIRCOUTCOMES.116.003552 PMC5540644

[14] Racism and Health. Centers for Disease Control and Prevention; 2023. https://www.cdc.gov/minorityhealth/racism-disparities/index.html#:~:text=The%20data%20show%20that%20racial,compared%20to%20their%20White%20counterparts.

[15] Rocca WA, Boyd CM, Grossardt BR, et al. Prevalence of multimorbidity in a geographically defined american population. Mayo Clin Proc. 2014;89(10):1336–49. 10.1016/j.mayocp.2014.07.010. 25220409 10.1016/j.mayocp.2014.07.010 PMC4186914

[16] How has our nation’s population changed? U.S. Census Bureau; 2023. https://www.census.gov/library/visualizations/interactive/how-has-our-nations-population-changed.html

[17] Most children younger than age 1 are minorities, census bureau reports. U.S. Census Bureau; 2012. https://www.census.gov/newsroom/releases/archives/population/cb12-90.html.

[18] *Overview of the national (nationwide) inpatient sample (NIS)*. Agency for Healthcare Research and Quality Accessed July 5, 2023. https://hcup-us.ahrq.gov/nisoverview.jsp

[19] *Trend weights for HCUP NIS data*. Accessed July 5, 2023. https://hcup-us.ahrq.gov/db/nation/nis/trendwghts.jsp

[20] *Clinical classifications software refineD (CCSR)*. Agency for Healthcare Research and Quality Accessed July 5, 2023. https://hcup-us.ahrq.gov/toolssoftware/ccsr/ccs_refined.jsp

[21] *Elixhauser comorbidity software refined for ICD-10-CM*. Agency for Healthcare Research and Quality Accessed July 5, 2023. https://hcup-us.ahrq.gov/toolssoftware/comorbidityicd10/comorbidity_icd10.jsp

[22] NIS DESCRIPTION OF DATA ELEMENTS. Agency for Healthcare Research and Quality. https://hcup-us.ahrq.gov/db/nation/nis/nisdde.jsp

[23] *Coronavirus disease 2019 (COVID-19)-associated hospitalization surveillance network (COVID-NET)*. Centers for Disease Control and Prevention Accessed July 5, 2023. https://www.cdc.gov/coronavirus/2019-ncov/covid-data/covid-net/purpose-methods.html

[24] *Explore census data*. U.S. Census Bureau Accessed July 5, 2023. https://data.census.gov/

[25] Gauthier J, Wu QV, Gooley TA. Cubic splines to model relationships between continuous variables and outcomes: a guide for clinicians. Bone Marrow Transplant. 2020;55(4):675–80. 10.1038/s41409-019-0679-x. 31576022 10.1038/s41409-019-0679-x

[26] Mackey K, Ayers CK, Kondo KK, et al. Racial and ethnic disparities in COVID-19–related infections, hospitalizations, and deaths: a systematic review. Ann Intern Med. 2021;174(3):362–73. 10.7326/M20-6306. 33253040 10.7326/M20-6306 PMC7772883

[27] A Mirajkar, A Oswald, M Rivera, et al. 2023 Racial disparities in patients hospitalized for COVID-19. *J Natl Med Assoc*. Published online June:S0027968423000810. 10.1016/j.jnma.2023.06.006 10.1016/j.jnma.2023.06.006 PMC10277852 37407381

[28] Risk* for COVID-19 infection, hospitalization, and death by race/ethnicity*. Centers for Disease Control and Prevention Accessed July 6, 2023. https://stacks.cdc.gov/view/cdc/105022

[29] *COVID-NET laboratory-confirmed COVID-19 hospitalizations*. Centers for Disease Control and Prevention Accessed December 11, 2023. https://covid.cdc.gov/covid-data-tracker/#covidnet-hospitalization-network

[30] Taylor CA, Whitaker M, Anglin O, et al. COVID-19–Associated hospitalizations among adults during SARS-CoV-2 delta and omicron variant predominance, by race/ethnicity and vaccination status — cOVID-NET, 14 States, July 2021–January 2022. MMWR Morb Mortal Wkly Rep. 2022;71(12):466–73. 10.15585/mmwr.mm7112e2. 35324880 10.15585/mmwr.mm7112e2 PMC8956338

[31] Jefferson C, Watson E, Certa JM, et al. Differences in COVID-19 testing and adverse outcomes by race, ethnicity, sex, and health system setting in a large diverse US cohort. PLoS ONE. 2022;17(11):e0276742. 10.1371/journal.pone.0276742. 36417366 10.1371/journal.pone.0276742 PMC9683575

[32] Price-Haywood EG, Burton J, Fort D, Seoane L. Hospitalization and mortality among black patients and white patients with Covid-19. N Engl J Med. 2020;382(26):2534–43. 10.1056/NEJMsa2011686. 32459916 10.1056/NEJMsa2011686 PMC7269015

[33] Wong MS, Haderlein TP, Yuan AH, Moy E, Jones KT, Washington DL. Time trends in racial/ethnic differences in COVID-19 infection and mortality. Int J Environ Res Public Health. 2021;18(9):4848. 10.3390/ijerph18094848. 34062806 10.3390/ijerph18094848 PMC8124342

[34] P Braveman, L Gottlieb. 2014 The social determinants of health: it’s time to consider the causes of the causes. *Public Health Rep Wash DC 1974* 129 Suppl 2(Suppl 2):19–31. 10.1177/00333549141291S206 10.1177/00333549141291S206 PMC3863696 24385661

[35] Rogers TN, Rogers CR, VanSant-Webb E, Gu LY, Yan B, Qeadan F. Racial disparities in COVID-19 mortality among essential workers in the United States. World Med Health Policy. 2020;12(3):311–27. 10.1002/wmh3.358. 32837779 10.1002/wmh3.358 PMC7436547

[36] N Goldman, AR Pebley, K Lee, T Andrasfay, 2020Pratt B. *Racial and ethnic differentials in COVID-19-related job exposures by occupational standing in *the* US*. Infectious Diseases (except HIV/AIDS); 10.1101/2020.11.13.20231431 10.1371/journal.pone.0256085 PMC8409606 34469440

[37] Schnake-Mahl AS, Lazo M, Dureja K, Ehtesham N, Bilal U. Racial and ethnic inequities in occupational exposure across and between US cities. SSM - Popul Health. 2021;16:100959. 10.1016/j.ssmph.2021.100959. 34805478 10.1016/j.ssmph.2021.100959 PMC8590507

[38] Sze S, Pan D, Nevill CR, et al. Ethnicity and clinical outcomes in COVID-19: a systematic review and meta-analysis. EClinicalMedicine. 2020;29–30:100630. 10.1016/j.eclinm.2020.100630. 10.1016/j.eclinm.2020.100630 PMC7658622 33200120

[39] Williams DR, Priest N, Anderson NB. Understanding associations among race, socioeconomic status, and health: patterns and prospects. Health Psychol. 2016;35(4):407–11. 10.1037/hea0000242. 27018733 10.1037/hea0000242 PMC4817358

[40] Williams DR, Mohammed SA, Leavell J, Collins C. Race, socioeconomic status, and health: complexities, ongoing challenges, and research opportunities. Ann N Y Acad Sci. 2010;1186:69–101. 10.1111/j.1749-6632.2009.05339.x. 20201869 10.1111/j.1749-6632.2009.05339.x PMC3442603

[41] Galea S, Tracy M, Hoggatt KJ, Dimaggio C, Karpati A. Estimated deaths attributable to social factors in the United States. Am J Public Health. 2011;101(8):1456–65. 10.2105/AJPH.2010.300086. 21680937 10.2105/AJPH.2010.300086 PMC3134519

[42] Daniel H, Bornstein SS, Kane GC, for the Health and Public Policy Committee of the American College of Physicians. Addressing social determinants to improve patient care and promote health equity: an American college of physicians position paper. Ann Intern Med. 2018;168(8):577. 10.7326/M17-2441. 29677265 10.7326/M17-2441

[43] Ahmad K, Chen EW, Nazir U, et al. Regional variation in the association of poverty and heart failure mortality in the 3135 counties of the United States. J Am Heart Assoc. 2019;8(18):e012422. 10.1161/JAHA.119.012422. 31480884 10.1161/JAHA.119.012422 PMC6818020

[44] Ashleigh Guadagnolo B, Cina Kristin, Helbig Petra, et al. Medical mistrust and less satisfaction with health care among native americans presenting for cancer treatment. J Health Care Poor Underserved. 2008;20(1):210–26. 10.1353/hpu.0.0108. 10.1353/hpu.0.0108 PMC2665798 19202258

[45] Skewes MC, Gonzalez VM, Gameon JA, et al. Health disparities research with American Indian communities: the importance of trust and transparency. Am J Community Psychol. 2020;66(3–4):302–13. 10.1002/ajcp.12445. 32652706 10.1002/ajcp.12445 PMC7772225

[46] A Mangla, N Agarwal. 2023 Clinical practice issues in American Indians and Alaska Natives. In: *StatPearls*. StatPearls Publishing Accessed December 17, 2023. http://www.ncbi.nlm.nih.gov/books/NBK570601/34033363

[47] Singh GK, Daus GP, Allender M, et al. Social determinants of health in the United States: addressing major health inequality trends for the nation, 1935–2016. Int J MCH AIDS. 2017;6(2):139–64. 10.21106/ijma.236. 29367890 10.21106/ijma.236 PMC5777389

[48] Saydah SH, Imperatore G, Beckles GL. Socioeconomic status and mortality. Diabetes Care. 2013;36(1):49–55. 10.2337/dc11-1864. 22933434 10.2337/dc11-1864 PMC3526248

[49] Hahn RA, Eaker E, Barker ND, Teutsch SM, Sosniak W, Krieger N. Poverty and death in the United States—1973 and1995. Epidemiology. 1995;6(5):490–7. 10.1097/00001648-199509000-00005. 8562624

[50] Knapp EA, Bilal U, Dean LT, Lazo M, Celentano DD. Economic insecurity and deaths of despair in US counties. Am J Epidemiol. 2019;188(12):2131–9. 10.1093/aje/kwz103. 31172197 10.1093/aje/kwz103 PMC7212405

