# Supplementary material for: Racial and Ethnic Disparities and the National Burden of COVID-19 on Inpatient Hospitalizations: A Retrospective Study in the United States in the Year 2020
Source: J Racial Ethn Health Disparities. 2024 Sep 24;12(4):2509–20. doi: 10.1007/s40615-024-02069-y (PMC12241180; doi:10.1007/s40615-024-02069-y)
Supplement: Supplementary file 1 — Supplementary file1 (DOCX 232 KB) [file 40615_2024_2069_MOESM1_ESM.docx]

**Supplementary Material:**

**Title:** Racial and Ethnic Disparities and the National Burden of COVID-19 on Inpatient Hospitalizations: A Retrospective Study in the United States in the Year 2020

**Journal name:** Journal of Racial and Ethnic Health Disparities

**Authors:** Amanda Nguyen MD,^1^ Russell G. Buhr MD PhD,^2,3^ Gregg C. Fonarow MD,^4^ Jeffrey J. Hsu MD PhD,^5^ Arleen F. Brown MD PhD,^6^ Boback Ziaeian MD PhD^4,5^

^1^Department of Medicine, UC Davis Medical Center, Sacramento, CA

^2^ Division of Pulmonary, Critical Care and Sleep Medicine, David Geffen School of Medicine at UCLA, Los Angeles, CA,

^3^Department of Medicine, Greater Los Angeles Veterans Affairs Health Care System, Los Angeles, CA

^4^ Division of Cardiology, David Geffen School of Medicine at UCLA, Los Angeles, CA ^5^Division of Cardiology, VA Greater Los Angeles Healthcare System, Los Angeles, CA,

^6^Division of General Internal Medicine and Health Services Research, Department of Medicine, David Geffen School of Medicine at UCLA, Los Angeles, CA

**Corresponding Author:**

Boback Ziaeian, M.D., Ph.D.

Division of Cardiology, University of California, Los Angeles

Division of Cardiology, VA Greater Los Angeles Healthcare System

1301 Wilshire Blvd, 111E

Los Angeles, CA 90073

Email: [BZiaeian@mednet.ucla.edu](mailto:BZiaeian@mednet.ucla.edu)

| **Supplementary Table 1: ICD-10, ICD-10-CM CCSR, and ICD-10-PCS CSSR Codes** | | |
| --- | --- | --- |
| **Comorbidities** | **ICD10** | **ICD-10-CM CCSR** |
| COVID-19 Hospitalization | U07.1 |  |
| Any heart Failure | I51.81, R57.0 | CIR019 |
| Any atrial fibrillation/flutter | I48.0 – I48.92 |  |
| Ventricular Tachycardia | I47.2 |  |
| Coronary artery disease |  | CIR011 |
| Cerebral Infarction/Stroke | G45.0 – G46.8; H34.00 – H34.239; I60.00 – I63.9;  I65.01 – I66.9; I69.30 – I69.398; I69.80 – I69.998;  P91.821 – P91.829; I69.331 – I69.369; I69.831 – I69.969 |  |
| Peripheral/Visceral Vascular Disease | A52.00 – A52.09; I70.0 – I75.89; I77.0 – I79.8; K55.1; Z95.820 – Z95.828 |  |
| Obesity | R93.9 | END009 |
| Dyslipidemia |  | END010 |
| Diabetes |  | END002 – END006, PRG019 |
| Hypertension |  | CIR007, CIR008, PRG020 |
| Asthma |  | RSP009 |
| COPD |  | RSP008 |
| Sleep apnea | G47.30, G47.31, G47.33, G47.36, G47.37, G47.39 |  |
| Chronic kidney disease | Z94.0, Z99.2, Z91.15 | GEN003 |
| Any liver failure |  | DIG018 |
| Chronic liver failure | K72.10, K72.11, K72.90, K72.91, K70.40, K70.41 |  |
| Thyroid disorders |  | END001 |
| Rheumatoid arthritis |  | MUS003 |
| Autoimmune disorders | A18.01 – A18.02; A39.84;  A54.41 – A54.42; L40.50 – L40.59; L90.0; L94.0 – L94.3; M05.00 – M05.79; M05.7A; M05.80 – M05.89; M05.8A; M05.9; M06.00 – M06.09;  M06.0A; M06.1 – M06.4; M06.80 – M06.89; M06.8A; M06.9; M07.60 – M08.09; M08.0A; M08.1 – M08.29; M08.2A; M08.3 – M08.48;  M08.4A; M08.80 – M08.99; M08.9A; M12.00 – M12.09; M30.0 – M35.09; M35.0A – M35.0C; M35.1 – M36.8; M45.0 – M45.9; M4.5A0 – M4.5A8; M4.5AB; M32.11 |  |
| HIV/AIDS | B20; O98.711 – O98.73; Z21 |  |
| Immunity Disorders/Long Term Immunosuppression | Z92.241, Z92.25, Z79.52 | BLD008 |
| Coagulopathy | D61.09 – D61.9; D65; D66; D67; D68.0 – D69.9; D75.82 – D75.84; O99.111 – O99.13 |  |
| Any hematologic cancer (ie Leukemia/lymphoma, multiple myeloma, myeloproliferative neoplasms) |  | NEO057 – NEO065, NEO068 |
| Any solid cancer |  | NEO001 – NEO025; NEO029 – NEO056; NEO066, NEO067, NEO069 – NEO072 |
| Secondary malignancy | C7B.00 – C7B.8; C80.0 | NEO070 |
| Tobacco use disorder | T65.211A – T65.294A; Z87.891 | MBD024 |
| Alcohol use disorder |  | MBD017 |
| Substance use disorder |  | MBD018 – MBD023; MBD025; MBD028 – MBD033 |
| **Acute complications** | **ICD10** | **ICD-10-CM CCSR** |
| Acute MI |  | CIR009 |
| Cardiac arrest |  | CIR018 |
| Acute renal failure |  | GEN002 |
| Acute liver failure | K72.00, K72.01 |  |
| Acute cerebrovascular events |  | CIR020 – CIR024 |
| Acute thromboembolic events, including PE |  | CIR013, CIR033 |
| Acute respiratory failure | J80, J96.00 – J96.92 |  |
| Shock |  | SYM003 |
| Critical illness myopathy/polyneuropathy | G62.81, G72.81 |  |
| Delirium/disorientation | F05, R41.0 |  |
| **Procedures** | **ICD10 PR** | **ICD-10-PCS CSSR** |
| PA Catheter |  | MAM003 |
| Cardiac catheterization |  | IMG001 |
| PCI |  | CAR004 |
| CPR |  | ESA005 |
| Pacemaker/ICD |  | CAR026 |
| Cardioversion |  | ESA006 |
| Vasopressor infusion |  | ADM006 |
| ECMO alone |  | ESA002 |
| MCS/ECMO |  | ESA002, ESA008, ESA009, ESA010, CAR027 |
| Noninvasive ventilation |  | ESA004 |
| Mechanical ventilation (including all mechanical ventilations, intubations and tracheostomies) |  | ESA003, RES007, RES010 |
| Thoracentesis/Thoracoscopic Pleural Drainage |  | RES003, RES005, RES006 |
| Bronchoscopy |  | RES001, RES004 |
| Peritoneal Dialysis |  | ADM013 |
| Any hemodialysis |  | ESA001 |
| CRRT | 5A1D90Z |  |
| Blood transfusion |  | ADM001 |

| **Supplementary Table 2: Primary COVID-19 Hospitalizations and Mortality, stratified by Median Income Among Different Race and Ethnic Groups, Unadjusted** | | | | | | |
| --- | --- | --- | --- | --- | --- | --- |
| **Median Income Quartile** | **White** | **Black** | **Hispanic** | **AAPI** | **Native American** | **Other** |
| COVID-19 Hospitalizations | n = 538,490 | n = 189,865 | n = 212,235 | n = 33,080 | n = 10,695 | n = 42,175 |
| Quartile 1 ($1 – 49,999) | 27.6% | 52.3% | 37.8% | 15.4% | 58.4% | 27.5% |
| Quartile 2 ($50,000 – 64,999) | 30.0% | 22.7% | 27.2% | 23.4% | 22.3% | 25.1% |
| Quartile 3 ($65,000 – 85,999) | 23.8% | 15.2% | 22.7% | 28.3% | 12.8% | 23.5% |
| Quartile 4 ($86,000+) | 18.7% | 9.8% | 12.2% | 32.9% | 6.5% | 23.8% |
| COVID-19 Mortality | n = 62,845 | n = 18,620 | n = 22,095 | n = 3,520 | n = 1,570 | n = 4,825 |
| Quartile 1 ($1 – 49,999) | 29.3% | 51.6% | 42.5% | 15.1% | 67.4% | 30.6% |
| Quartile 2 ($50,000 – 64,999) | 29.5% | 23.3% | 26.4% | 24.1% | 21.2% | 26.8% |
| Quartile 3 ($65,000 – 85,999) | 22.8% | 13.9% | 20.9% | 28.1% | 6.9% | 22.4% |
| Quartile 4 ($86,000+) | 18.4% | 11.1% | 10.2% | 32.6% | 4.5% | 20.3% |

| **Supplementary Table 3: Baseline Patient Characteristics/Demographics and Hospital Characteristics for Secondary COVID-19, by Race and Ethnicity** | | | | | | | |
| --- | --- | --- | --- | --- | --- | --- | --- |
| **Characteristic (%)** | **All Patients** | **White** | **Black** | **Hispanic** | **AAPI** | **Native American** | **Other** |
|  | n = 620,180^a^ | n = 287,235 | n = 120,915 | n = 141,210 | n = 19,755 | n = 6,070 | n = 28,065 |
| Median age, years (IQR 25%, 75%) | 63 (45, 76) | 70 (57, 81) | 61 (45, 72) | 52 (36, 67) | 64 (48, 77) | 54 (38, 67) | 58 (40, 72) |
| **Sex** | | | | | | | |
| Female | 49.3% | 48.2% | 51.9% | 50.1% | 48.7% | 51.9% | 46.9% |
| **Median Income Quartile** | | | | | | | |
| Quartile 1 ($1 – 49,999) | 34.0% | 26.3% | 50.2% | 38.6% | 16.1% | 55.9% | 27.2% |
| Quartile 2 ($50,000 – 64,999) | 26.4% | 28.2% | 22.8% | 26.4% | 22.4% | 24.3% | 24.3% |
| Quartile 3 ($65,000 – 85,999) | 22.6% | 24.6% | 16.3% | 23.2% | 28.9% | 12.8% | 23.9% |
| Quartile 4 ($86,000+) | 17.1% | 20.9% | 10.7% | 11.8% | 32.5% | 7.0% | 24.5% |
| **Insurance Status** | | | | | | | |
| Medicare | 47.1% | 59.8% | 45.2% | 27.5% | 42.1% | 34.3% | 33.5% |
| Medicaid | 20.2% | 10.2% | 22.9% | 35.4% | 21.2% | 37.9% | 28.6% |
| Private Insurance | 24.0% | 23.4% | 24.0% | 23.9% | 31.5% | 17.8% | 26.0% |
| Self-pay or No Charge | 4.7% | 2.6% | 4.1% | 9.2% | 2.6% | 2.8% | 7.3% |
| Other | 4.0% | 4.0% | 3.8% | 4.0% | 2.6% | 7.2% | 4.6% |
| **Hospital Location/Teaching status** | | | | | | | |
| Rural | 6.4% | 9.6% | 4.9% | 1.8% | 1.0% | 13.4% | 2.3% |
| Urban, nonteaching | 17.3% | 19.3% | 13.5% | 17.7% | 17.7% | 11.4% | 15.2% |
| Urban, teaching | 76.3% | 71.1% | 81.5% | 80.5% | 81.3% | 75.2% | 82.5% |
| **Hospital Division** | | | | | | | |
| New England | 4.0% | 5.1% | 2.9% | 3.3% | 2.5% | 0.74% | 2.5% |
| Middle Atlantic | 15.6% | 14.6% | 17.0% | 13.9% | 20.3% | 2.5% | 31.9% |
| East North Central | 14.4% | 18.9% | 15.5% | 6.4% | 9.4% | 5.4% | 6.9% |
| West North Central | 6.2% | 8.3% | 4.2% | 1.9% | 4.8% | 13.8% | 2.3% |
| South Atlantic | 19.6% | 18.1% | 32.0% | 15.2% | 8.8% | 5.7% | 17.4% |
| East South Central | 6.0% | 8.0% | 9.0% | 1.4% | 1.4% | 1.9% | 1.6% |
| West South Central | 14.3% | 12.4% | 12.4% | 20.7% | 7.5% | 11.7% | 16.9% |
| Mountain | 7.1% | 7.1% | 2.3% | 9.4% | 7.5% | 49.8% | 6.9% |
| Pacific | 12.9% | 7.4% | 4.8% | 27.8% | 37.8% | 8.5% | 13.6% |
| **Hospital Size** | | | | | | | |
| Small | 21.9% | 23.7% | 21.6% | 18.9% | 19.4% | 18.5% | 18.2% |
| Medium | 29.1% | 28.3% | 28.7% | 30.2% | 26.9% | 22.7% | 35.1% |
| Large | 49.1% | 48.0% | 49.7% | 50.9% | 53.7% | 58.8% | 46.7% |
| **Comorbidities** | | | | | | | |
| Any heart failure | 18.1% | 21.7% | 20.2% | 10.9% | 14.8% | 14.9% | 13.4% |
| Arrhythmia |  |  |  |  |  |  |  |
| Atrial fibrillation/flutter | 17.2% | 24.2% | 12.5% | 8.6% | 15.2% | 9.5% | 11.9% |
| Ventricular tachycardia | 2.4% | 2.5% | 2.9% | 1.6% | 2.0% | 2.4% | 2.1% |
| Coronary artery disease | 19.1% | 24.6% | 17.0% | 11.6% | 17.9% | 12.7% | 13.9% |
| Any cerebrovascular disease | 6.9% | 7.1% | 9.2% | 4.5% | 8.6% | 4.6% | 6.1% |
| Peripheral vascular disease | 4.7% | 6.0% | 4.1% | 3.0% | 4.8% | 3.1% | 2.8% |
| Obesity | 23.5% | 22.0% | 27.4% | 25.5% | 13.3% | 25.4% | 19.4% |
| Dyslipidemia | 35.0% | 40.8% | 33.3% | 26.4% | 39.0% | 23.7% | 28.5% |
| Diabetes | 38.8% | 35.2% | 44.4% | 40.5% | 45.8% | 44.8% | 38.0% |
| Hypertension | 35.1% | 36.0% | 37.2% | 31.9% | 37.7% | 29.1% | 32.1% |
| Asthma | 6.6% | 5.8% | 8.8% | 6.6% | 6.8% | 9.9% | 6.0% |
| COPD | 12.7% | 18.1% | 11.3% | 4.8% | 8.4% | 7.4% | 7.5% |
| Sleep apnea | 7.1% | 9.3% | 7.2% | 3.9% | 4.0% | 5.4% | 4.0% |
| Chronic renal failure | 22.4% | 23.3% | 30.2% | 15.6% | 22.7% | 17.3% | 17.1% |
| Chronic liver failure | 0.79% | 0.85% | 0.50% | 0.91% | 0.40% | 3.1% | 0.80% |
| Thyroid disorders | 12.3% | 17.0% | 7.1% | 8.2% | 9.7% | 8.5% | 9.0% |
| Rheumatoid arthritis | 1.6% | 2.0% | 1.3% | 1.1% | 1.0% | 2.1% | 1.1% |
| Autoimmune Disorders | 2.7% | 3.3% | 2.5% | 1.9% | 1.7% | 3.5% | 2.0% |
| HIV/AIDS | 0.69% | 0.30% | 1.8% | 0.59% | 0.33% | 0.33% | 0.89% |
| Immunity disorder/long-term  immunosuppression | 2.5% | 2.9% | 2.6% | 1.8% | 1.7% | 1.4% | 1.7% |
| Coagulopathy | 16.1% | 16.2% | 15.9% | 15.6% | 19.2% | 20.1% | 16.1% |
| Hematologic cancer | 1.7% | 2.3% | 1.3% | 1.2% | 1.6% | 0.33% | 1.3% |
| Solid cancer | 4.7% | 5.3% | 4.9% | 3.6% | 4.3% | 3.7% | 3.9% |
| Tobacco use disorder | 16.3% | 20.8% | 14.7% | 10.6% | 12.4% | 13.9% | 10.2% |
| Alcohol use disorder | 3.2% | 3.2% | 2.7% | 3.2% | 1.3% | 15.2% | 3.3% |
| Substance use disorder | 3.7% | 3.7% | 4.7% | 3.2% | 1.4% | 8.2% | 3.5% |

^a^N reported with appropriate NIS trend weights

| **Supplementary Table 4: Hospital Procedures, Complications and Outcomes for Secondary COVID-19 by Race and Ethnicity, Unadjusted (Age-Adjusted)^a^** | | | | | | | |
| --- | --- | --- | --- | --- | --- | --- | --- |
| **Characteristic** | **Overall population** | **White** | **Black** | **Hispanic** | **AAPI** | **Native American** | **Other** |
|  | n = 620,180^a^ | n = 287,235 | n = 120,915 | n = 141,210 | n = 19,755 | n = 6,070 | n = 28,065 |
| **Complications** | | | | | | | |
| Acute MI | 6.3% (2.8%) | 6.8% (2.6%) | 5.9% (2.9%) | 5.2% (2.9%) | 7.1% (3.3%) | 6.0% (3.5%) | 6.3% (3.2%) |
| Cardiac arrest | 3.1% (1.8%) | 2.4% (1.4%) | 3.8% (2.2%) | 3.6% (2.1%) | 3.6% (1.8%) | 2.7% (1.7%) | 3.6% (2.2%) |
| Acute renal failure | 34.0% (18.8%) | 35.2% (16.9%) | 41.8% (25.6%) | 25.5% (16.3%) | 33.8% (17.5%) | 29.9% (22.2%) | 31.6% (18.3%) |
| Acute liver failure | 1.7% (1.2%) | 1.4% (1.1%) | 1.9% (1.3%) | 1.8% (1.2%) | 2.4% (1.3%) | 2.6% (1.8%) | 2.1% (1.4%) |
| Acute cerebrovascular event | 3.9% (2.3%) | 4.2% (2.1%) | 4.0% (2.4%) | 2.9% (2.2%) | 4.9% (2.3%) | 2.6% (1.5%) | 3.9% (2.2%) |
| Acute thromboembolic events | 5.6% (3.8%) | 5.7% (3.7%) | 6.6% (4.5%) | 4.7% (3.5%) | 4.7% (3.1%) | 4.7% (3.5%) | 5.2% (3.6%) |
| Acute respiratory failure | 46.6% (31.2%) | 44.8% (26.9%) | 45.0% (30.7%) | 48.9% (35.5%) | 56.9% (39.2%) | 53.1% (40.6%) | 50.0% (34.1%) |
| Shock | 3.2% (2.7%) | 2.9% (2.3%) | 3.5% (3.6%) | 3.2% (2.5%) | 4.0% (2.3%) | 4.2% (3.2%) | 3.5% (2.5%) |
| Critical illness myopathy/neuropathy | 1.0% (0.68%) | 0.93% (0.58%) | 1.1% (0.69%) | 1.0% (0.71%) | 1.2% (0.84%) | 1.7% (1.3%) | 1.1% (0.71%) |
| Delirium/disorientation | 2.6% (1.3%) | 3.3% (1.3%) | 2.3% (1.1%) | 1.6% (1.1%) | 2.8% (2.0%) | 1.5% (1.0%) | 2.7% (1.8%) |
| **Hospital Procedures** | | | | | | | |
| Cardiac catheterization | 1.0% (0.60%) | 1.2% (0.69%) | 0.79% (0.52%) | 0.82% (0.55%) | 0.63% (0.39%) | 1.3% (0.76%) | 0.89% (0.58%) |
| CPR | 2.2% (1.3%) | 1.6% (0.88%) | 3.2% (1.8%) | 2.6% (1.6%) | 2.5% (1.3%) | 1.5% (0.88%) | 2.6% (1.4%) |
| Cardioversion | 0.64% (0.37%) | 0.62% (0.31%) | 0.71% (0.45%) | 0.59% (0.36%) | 0.58% (0.26%) | 0.58% (0.35%) | 0.73% (0.57%) |
| Vasopressor infusion | 4.1% (2.6%) | 3.6% (2.0%) | 4.6% (2.9%) | 4.1% (2.9%) | 5.9% (3.9%) | 3.3% (2.2%) | 5.3% (3.2%) |
| MCS/ECMO | 0.53% (0.67%) | 0.48% (0.83%) | 0.41% (0.46%) | 0.64% (0.66%) | 0.58% (0.51%) | 1.0% (0.73%) | 0.53% (0.63%) |
| Noninvasive ventilation | 6.2% (3.9%) | 6.5% (3.4%) | 5.7% (4.1%) | 5.8% (4.2%) | 8.2% (5.5%) | 4.6% (3.3%) | 6.0% (3.5%) |
| Any Mechanical Ventilation^b^ | 17.1% (12.0%) | 14.8% (10.2%) | 19.0% (13.4%) | 18.4% (13.3%) | 20.6% (14.0%) | 26.7% (20.9%) | 20.4% (13.2%) |
| Thoracentesis/  thoracoscopic pleural drainage | 2.0% (1.5%) | 2.0% (1.4%) | 1.6% (1.4%) | 2.2% (1.8%) | 1.7% (1.4%) | 2.5% (1.7%) | 2.8% (2.0%) |
| Bronchoscopy | 1.5% (1.3%) | 1.3% (1.4%) | 1.5% (1.1%) | 1.8% (1.5%) | 1.7% (1.2%) | 3.5% (2.8%) | 1.7% (1.2%) |
| Any hemodialysis | 6.6% (4.3%) | 4.4% (2.8%) | 11.3% (6.8%) | 6.9% (4.6%) | 8.6% (5.2%) | 8.7% (6.0%) | 6.4% (4.0%) |
| CRRT | 1.2% (0.85%) | 0.90% (0.67%) | 1.9% (1.1%) | 1.1% (0.77%) | 1.1% (0.73%) | 2.4% (1.8%) | 1.2% (0.89%) |
| Blood transfusion | 6.0% (4.8%) | 5.1% (3.5%) | 8.0% (6.7%) | 5.7% (4.6%) | 7.3% (6.1%) | 3.9% (3.2%) | 6.4% (4.7%) |
| **Hospital Stay Characteristics** | | | | | | | |
| Mean length of stay (95% CI) | 9.0 (8.8 – 9.1) | 8.5 (8.4 – 8.6) | 9.7 (9.5 – 9.9) | 9.0 (8.8 – 9.2) | 9.6 (9.0 – 10.1) | 9.5 (8.8 – 10.2) | 9.6 (9.2 – 10.0) |
| Mean cost of stay (95% CI) | $ 25,884 ($ 25,282 – $26,485) | $22,143 ($21,642  – $22,644) | $ 26,427 ($25,493  – $27,360) | $ 30,443 ($29,201– $31,685) | $ 34,603 ($32,068  – $37,138 ) | $ 32,430  ($28,446  – $36,414) | $30,391  ($28,504 – $32,279) |
| **Disposition** | | | | | | | |
| Home/Routine | 45.5% (71.5%) | 37.2% (64.4%) | 45.6% (61.8%) | 60.6% (68.6%) | 47.1% (67.2%) | 48.4% (56.3%) | 52.5% (65.7%) |
| Transfer to short-  term hospital | 3.1% (2.9%) | 3.4% (3.4%) | 2.9% (2.9%) | 2.5% (2.5%) | 3.1% (3.7%) | 4.8% (4.2%) | 3.0% (2.9%) |
| Transfer to skilled  nursing facility | 21.3% (9.9%) | 27.2% (13.4%) | 21.7% (13.8%) | 10.9% (8.0%) | 16.7% (9.6%) | 17.1% (14.1%) | 15.2% (9.8%) |
| Home health care | 11.7% (7.0%) | 12.8% (8.4%) | 12.2% (9.5%) | 9.5% (8.3%) | 12.2% (8.5%) | 8.7% (7.3%) | 10.3% (8.5%) |
| Against medical advice | 1.3% (1.5%) | 1.1% (2.3%) | 1.8% (2.5%) | 1.3% (1.6%) | 0.86% (1.1%) | 2.1% (3.2%) | 1.3% (1.9%) |
| Died in hospital | 17.0% (7.1%) | 18.0% (8.0%) | 15.7% (9.5%) | 15.2% (11.0%) | 19.9% (9.9%) | 19.0% (14.9%) | 17.7% (11.2%) |

^a^age-adjusted rates reported in parentheses

^b^Any mechanical ventilation defined as combined mechanical ventilation, intubation, and tracheostomy

| **Supplementary Table 5: Disparity Measures of the Age-Standardized Hospitalization Rate for Secondary COVID-19 by Sex, and Race and Ethnicity** | | | |
| --- | --- | --- | --- |
|  | **Overall population** | **Male** | **Female** |
| **Total Secondary COVID-19 Hospitalization Rate^a^** | | | |
| Crude Hospitalization Rate | 187.0 | 193.1 | 181.2 |
| Age-Standardized  Hospitalization Rate (95% CI) | 164.6 (164.2 – 165.0) | 177.6 (176.9 – 178.2) | 155.9 (155.3 – 156.5) |
| **White** | | | |
| Crude Hospitalization Rate | 149.8 | 157.3 | 142.4 |
| Age-Standardized  Hospitalization Rate (95% CI) | 109.6 (109.2 – 110.1) | 119.6 (119.0 – 120.2) | 102.6 (102.0 – 103.2) |
| **Black** | | | |
| Crude Hospitalization Rate | 302.6 | 305.6 | 299.8 |
| Age-Standardized  Hospitalization Rate (95% CI) | 301.2 (299.5 – 303.0) | 332.0 (329.1 – 334.9) | 282.2 (279.9 – 284.4) |
| Standardized Rate Ratio (95%  CI )^b^ | 2.75 (2.73 – 2.77) | 2.78 (2.75 – 2.80) | 2.75 (2.72 – 2.78) |
| Excess (95% CI )^b^ | 191.60 (189.80 – 193.40) | 212.40 (209.46 – 215.33) | 179.57 (177.23 – 181.91) |
| **Hispanic** | | | |
| Crude Hospitalization Rate | 227.4 | 227.9 | 226.8 |
| Age-Standardized  Hospitalization Rate (95% CI ) | 275.7 (274.2 – 277.2) | 312.5 (310.0 – 315.0) | 250.9 (249.0 – 252.8) |
| Standardized Rate Ratio (95%  CI ) | 2.51 (2.50 – 2.53) | 2.61 (2.59 – 2.64) | 2.45 (2.42 – 2.47) |
| Excess (95% CI ) | 166.06 (164.50 – 167.63) | 192.93 (190.36 – 195.49) | 148.28 (146.29 – 150.28) |
| **AAPI** | | | |
| Crude Hospitalization Rate | 97.5 | 104.9 | 90.7 |
| Age-Standardized  Hospitalization Rate (95% CI ) | 96.0 (94.7 – 97.4) | 111.9 (109.7 – 114.2) | 84.8 (83.0 – 86.5) |
| Standardized Rate Ratio (95%  CI ) | 0.88 (0.86 – 0.89) | 0.94 (0.92 – 0.96) | 0.83 (0.81 – 0.84) |
| Excess (95% CI ) | -13.59 (-15.02 – -12.16) | -7.71 (-10.04 – -5.37) | -17.83 (-19.65 – -16.01) |
| **Native American** | | | |
| Crude Hospitalization Rate | 269.6 | 263.1 | 275.9 |
| Age-Standardized  Hospitalization Rate (95% CI ) | 278.5 (271.3 – 285.8) | 282.8 (272.1 – 293.8) | 278.1 (268.2 – 288.3) |
| Standardized Rate Ratio (95%  CI ) | 2.54 (2.47 – 2.61) | 2.36 (2.28 – 2.46) | 2.71 (2.61 – 2.81) |
| Excess (95% CI ) | 168.86 (161.59 – 176.12) | 163.20 (152.37 – 174.02) | 175.54 (165.55 – 185.54) |
| **Other** | | | |
| Crude Hospitalization Rate | 184.1 | 200.7 | 168.1 |
| Age-Standardized  Hospitalization Rate (95% CI ) | 247.4 (244.4 – 250.5) | 299.0 (293.8 – 304.3) | 208.2 (204.5 – 212.0) |
| Standardized Rate Ratio (95%  CI ) | 2.26 (2.23 – 2.29) | 2.50 (2.45 – 2.55) | 2.03 (1.99 – 2.07) |
| Excess (95% CI ) | 137.80 (134.68 – 140.92) | 179.41 (174.11 – 184.71) | 105.62 (101.84 – 109.40) |

^a^ Directly standardized rates (per 100,000)

^b^Values are presented as ratios or excess number of admissions per 100,000. Rate defined as ratio of age-standardized hospitalization rate of subgroup over reference (White). Excess is defined as absolute difference of age-standardized hospitalization rate between subgroup and reference (White).

| **Supplementary Table 6: Patient Factors Associated with Inpatient Mortality for Secondary COVID-19 Hospitalizations** | | | |
| --- | --- | --- | --- |
| **Characteristic** | **OR** | **CI** | **P-value** |
| Age | See spline graph^a^ |  |  |
| Female Sex | 0.77 | 0.75 – 0.80 | < 0.001 |
| **Race (relative to White)** | | | |
| Black | 0.99 | 0.93 – 1.04 | 0.589 |
| Hispanic | 1.31 | 1.24 – 1.39 | < 0.001 |
| AAPI | 1.24 | 1.13 – 1.36 | < 0.001 |
| Native American | 1.83 | 1.55 – 2.16 | < 0.001 |
| Other | 1.27 | 1.16 – 1.39 | < 0.001 |
| **Insurance (relative to Medicare)** | | | |
| Medicaid | **1.20** | **1.12 – 1.29** | **< 0.001** |
| Private Insurance | 1.16 | 1.08 – 1.23 | < 0.001 |
| Self-pay or No Charge | 1.27 | 1.13 – 1.44 | < 0.001 |
| Other | 2.38 | 2.12 – 2.68 | < 0.001 |
| **Median Income Quartile (relative to Quartile 1 ($1-49,999)** | | | |
| Quartile 2 ($50,000 – 64,999) | 0.88 | 0.84 – 0.93 | < 0.001 |
| Quartile 3 ($65,000 – 85,999) | 0.82 | 0.77 – 0.87 | < 0.001 |
| Quartile 4 ($86,000+) | 0.74 | 0.70 – 0.79 | < 0.001 |
| **Hospital location/teaching status (relative to rural)** |  |  |  |
| Urban, nonteaching | 1.35 | 1.22 – 1.49 | < 0.001 |
| Urban, teaching | 1.37 | 1.25 – 1.50 | < 0.001 |
| **Hospital division (relative to New England)** | | | |
| Middle Atlantic | 1.26 | 1.09 – 1.44 | 0.001 |
| East North Central | 1.02 | 0.89 – 1.18 | 0.776 |
| West North Central | 1.10 | 0.94 – 1.29 | 0.218 |
| South Atlantic | 1.16 | 1.01 – 1.33 | 0.033 |
| East South Central | 1.42 | 1.20 – 1.66 | < 0.001 |
| West South Central | 1.35 | 1.17 – 1.57 | < 0.001 |
| Mountain | 1.33 | 1.14 – 1.54 | < 0.001 |
| Pacific | 1.39 | 1.21 – 1.60 | < 0.001 |
| **Month of admission (relative to December)** | | | |
| November | 1.00 | 0.95 – 1.06 | 0.968 |
| October | 0.91 | 0.84 – 0.98 | 0.011 |
| September | 0.87 | 0.80 – 0.94 | < 0.001 |
| August | 0.91 | 0.84 – 0.98 | 0.009 |
| July | 1.05 | 0.97 – 1.12 | 0.210 |
| June | 0.97 | 0.89 – 1.05 | 0.446 |
| May | 1.16 | 1.07 – 1.26 | < 0.001 |
| April | 2.10 | 1.96 – 2.24 | < 0.001 |
| March | 2.72 | 2.47 – 2.99 | < 0.001 |
| **Comorbid Conditions** | | | |
| Heart Failure | 1.14 | 1.09 – 1.20 | < 0.001 |
| Atrial fibrillation/flutter | 1.23 | 1.18 – 1.29 | < 0.001 |
| Ventricular Tachycardia | 2.40 | 2.19 – 2.63 | < 0.001 |
| Cerebrovascular disease | 1.12 | 1.05 – 1.19 | < 0.001 |
| Coagulopathy | 1.97 | 1.88 – 2.06 | < 0.001 |
| Obesity | 1.26 | 1.20 – 1.32 | < 0.001 |
| Diabetes mellitus | 1.07 | 1.03 – 1.11 | < 0.001 |
| Hypertension | 0.78 | 0.75 – 0.82 | < 0.001 |
| COPD | 1.27 | 1.21 – 1.33 | < 0.001 |
| Asthma | 0.81 | 0.75 – 0.88 | < 0.001 |
| Tobacco use disorder | 0.72 | 0.69 – 0.76 | < 0.001 |
| Alcohol use disorder | 0.72 | 0.64 – 0.81 | < 0.001 |
| Drug related disorders | 0.66 | 0.58 – 0.75 | < 0.001 |
| Chronic kidney disease | 1.09 | 1.04 – 1.14 | < 0.001 |
| Chronic liver disease | 2.37 | 2.01– 2.79 | < 0.001 |
| HIV/AIDS | 0.97 | 0.76 – 1.24 | 0.808 |
| Immunity disorder/long-term immunosuppression | 1.11 | 1.00 – 1.24 | 0.048 |
| Autoimmune disorders | 1.07 | 0.97 – 1.18 | 0.167 |

^a^see Supplementary Figure 2 for the spline model for age

Supplementary Fig. 1 Figure showing number of primary COVID-19 admissions and mortality rate, by month

^a^Mortality rate defined as the number of primary COVID-19 deaths/total number of primary COVID-19 admissions

Supplementary Fig. 2 Age spline model, showing non-linear relationship between age and odds of inpatient mortality


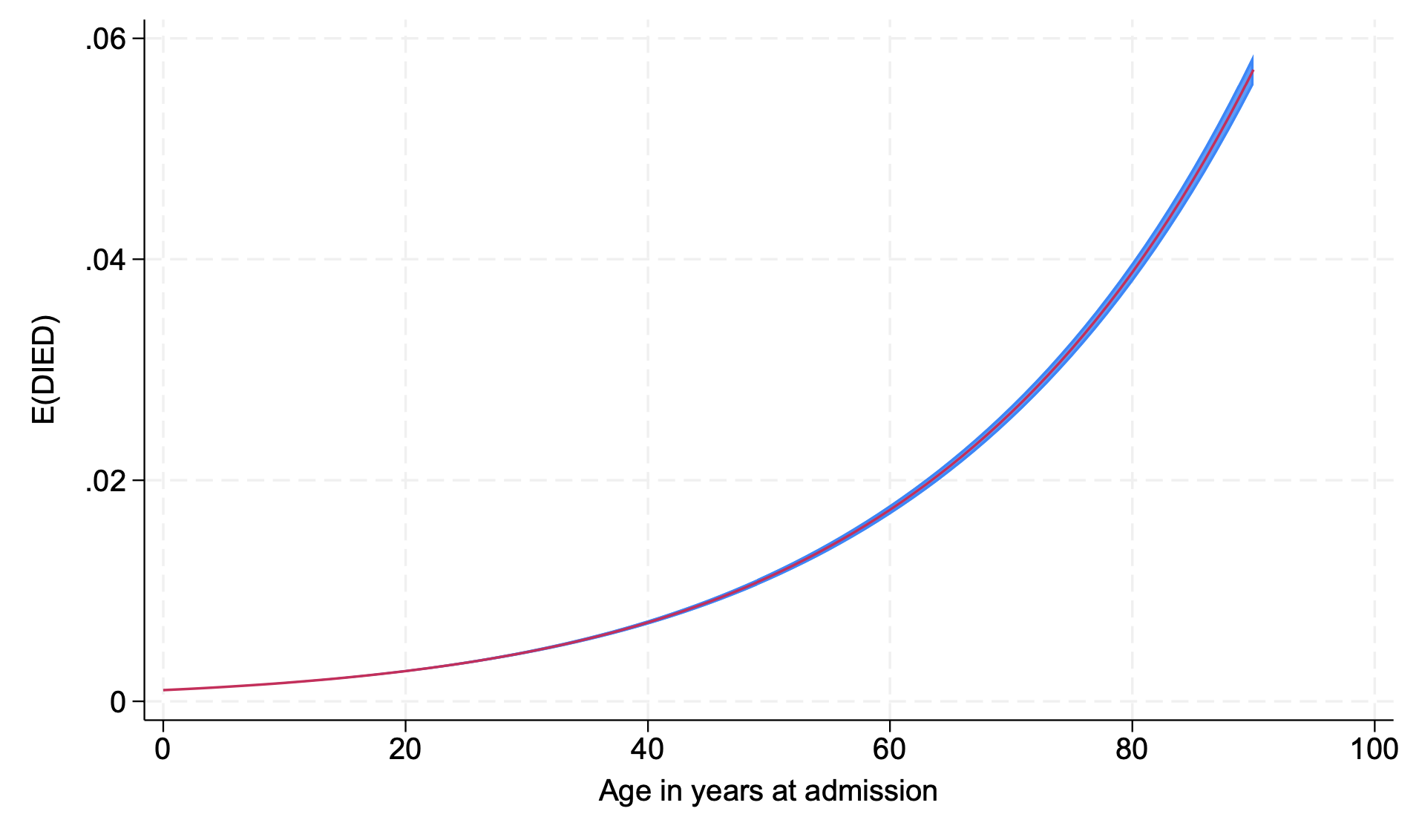


Supplementary Fig. 3 Figure showing number of secondary COVID-19 admissions and mortality rate, by month

^a^Mortality rate defined as the number of secondary COVID-19 deaths/total number of secondary COVID-19 admissions
